# *In-silico *identification of phenotype-biased functional modules

**DOI:** 10.1186/1477-5956-10-S1-S2

**Published:** 2012-06-21

**Authors:** Kanchana Padmanabhan, Kevin Wilson, Andrea M Rocha, Kuangyu Wang, James R Mihelcic, Nagiza F Samatova

**Affiliations:** 1 Department of Computer Science, North Carolina State University, Raleigh, 27695, USA; 2 Computer Science and Mathematics Division, Oak Ridge National Laboratory, Oak Ridge, 37831, USA; 3 Department of Civil and Environmental Engineering, University of South Florida, Tampa, 33620, USA; 4 Bioinformatics Research Center, North Carolina State University, Raleigh, NC 27695, USA; 5 RTI International, Durham, NC 27709, USA

## Abstract

**Background:**

Phenotypes exhibited by microorganisms can be useful for several purposes, e.g., ethanol as an alternate fuel. Sometimes, the target phenotype maybe required in combination with other phenotypes, in order to be useful, for e.g., an industrial process may require that the organism survive in an anaerobic, alcohol rich environment and be able to feed on both hexose and pentose sugars to produce ethanol. This combination of traits may not be available in any existing organism or if they do exist, the mechanisms involved in the phenotype-expression may not be efficient enough to be useful. Thus, it may be required to genetically modify microorganisms. However, before any genetic modification can take place, it is important to identify the underlying cellular subsystems responsible for the expression of the target phenotype.

**Results:**

In this paper, we develop a method to identify statistically significant and phenotypically-biased functional modules. The method can compare the organismal network information from hundreds of phenotype expressing and phenotype non-expressing organisms to identify cellular subsystems that are more prone to occur in phenotype-expressing organisms than in phenotype non-expressing organisms. We have provided literature evidence that the phenotype-biased modules identified for phenotypes such as hydrogen production (dark and light fermentation), respiration, gram-positive, gram-negative and motility, are indeed phenotype-related.

**Conclusion:**

Thus we have proposed a methodology to identify phenotype-biased cellular subsystems. We have shown the effectiveness of our methodology by applying it to several target phenotypes. The code and all supplemental files can be downloaded from (http://freescience.org/cs/phenotype-biased-biclusters/).

## Background

Phenotypes that certain microorganisms express includes breaking down the lignocellulosic barrier of biomass, biodegradation of various environmental contaminants etc. Tackling problems in the areas of biore-mediation and bioenergy with the help of genetic engineering requires as a first step identifying the cellular subsystems that are involved in the phenotype-expression by an organism. The phenotype-related cellular subsystems may be detected using laboratory experimentation. However, to supplement experimentation methods, computational methodologies need to be used.

Biological relationships (e.g., protein functional associations) between proteins are often modeled as networks (*functional association networks from STRING *[1]), where each node is a protein and every pair of functionally associated proteins is connected with an edge. Functional association between proteins is derived from a number of clues like experimental data, gene-fusion, co-occurrence of the corresponding genes on the same operon, etc. The subgraphs of these networks can model the cellular subsystems.

Evolutionary conservation of cellular subsystems can be used as one clue to identify the phenotype-related cellular subsystems [2]. The cellular systems associated with a phenotype are more likely to be present across phenotype-expressing organisms and are less likely to be present across phenotype non-expressing organisms [2]. This strategy can be utilized to identify cellular subsystems that are likely phenotype-related.

An earlier work by Schmidt *et al *[2] focused on identifying phenotype-related functional modules that were modeled as cliques. Functional modules that have a clique structure require that every pair of proteins in the module has an edge between them. The density of the subgraph modeled as a clique is 1. Density is the ratio of the number of edges in the subgraph to the total number of possible edges in the subgraph. This method was one of the first to identify phenotype-related subgraphs. However, their subgraph identification condition is too stringent to model all biological functional modules. This is primarily because biological networks are prone to missing information (like missing edges) [3]. Paccanarot *et al *[4] explain that most of the errors in the networks are false-negatives, i.e., edges that were not predicted. Hence, Schmidt *et al *[2] method may not identify the complete phenotype-related cellular subsystems. They acknowledge this as a drawback and use the identified cliques as input into another algorithm called DENSE [5], that can extract extended subsystems from a single organismal network. These subsystems may or may not be related towards the target phenotype.

Additionally, Schmidt *et al *[2] method requires two inputs: the parameter *α*-the least number of phenotype-expressing organisms the identified clique has to be present in and the parameter *β*-the number of phenotype non-expressing organisms the identified clique can be present in. These parameters may be hard to estimate beforehand and, hence, multiple runs with different parameter values may be required.

Spirin *et al *[6] showed that significantly dense "non-clique" clusters formed biologically relevant functional modules. They provide an example of a functional module associated with *cell-cycle regulation *consisting of cyclins (*CLB1-4 *and *CLN2*), cyclin-dependent kinases (*CKS1 *and *CDC28*), and a nuclear import protein *NIP29 *identified from *Saccharomyces cerevisiae *network that is not a clique. Hwang *et al *[7] showed that maximal clique enumeration methods discard over 90% of network nodes when applied to the PPI network of *Saccharomyces cerevisiae*. Hendrix *et al *[5] identified "non-clique" functional modules that were verified by literature. Habibi *et al *[8] showed that protein complexes that are usually thought to be cliques could also have different topologies (MIPS ID: 510.40.10 and 550.1.213 complexes) and this could primarily be due to the fact that biological data sources contain noise and possibly do not contain the entire information due to limitations of experiments. Additionally, their detailed study of the densities of the existing protein complexes from various sources [9-13] has revealed that many complexes have density less than 0.1.

**Figure 1:**
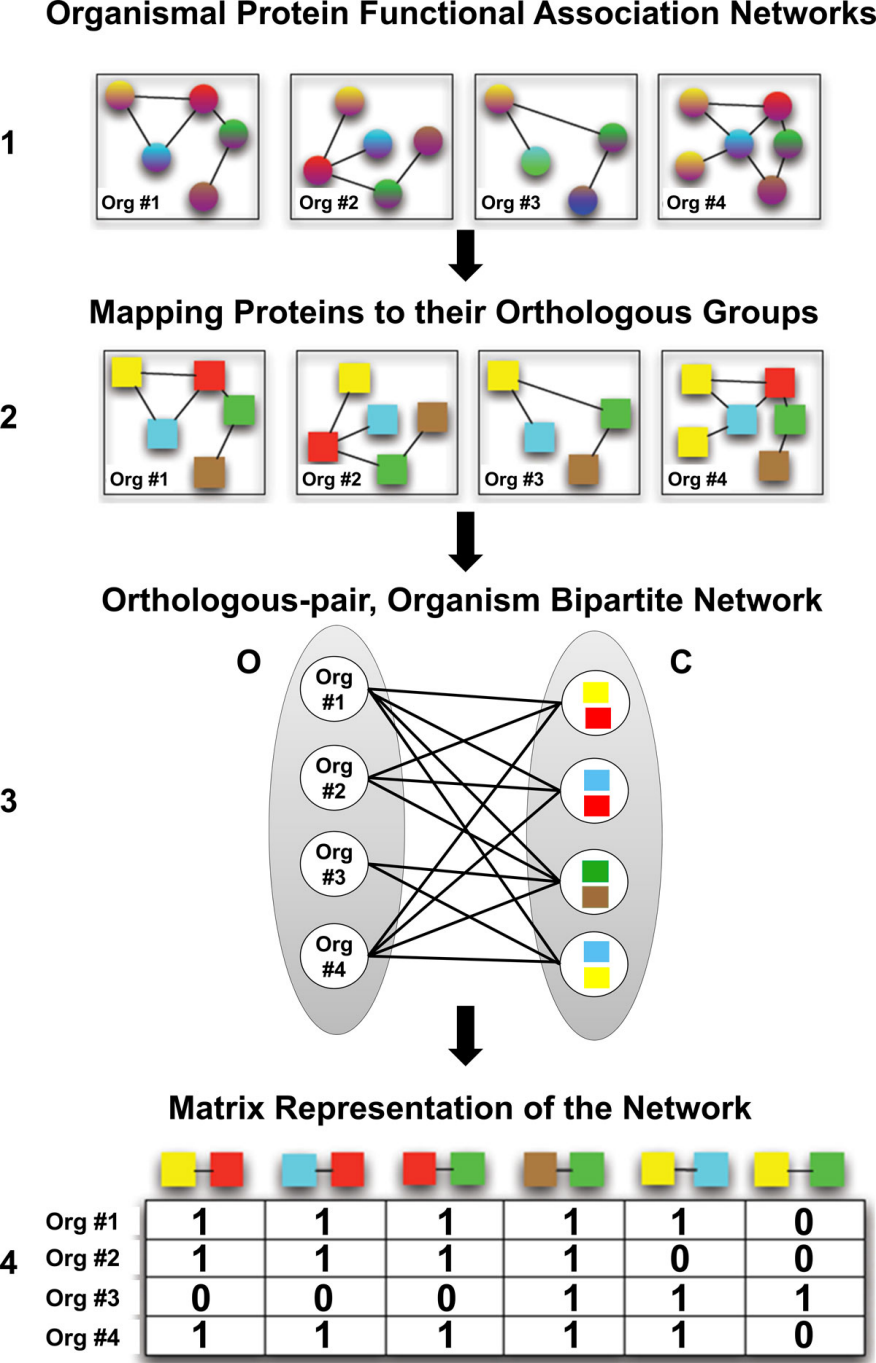
**Building the orthologous group-pair, organism bipartite network**.

**Figure 2:**
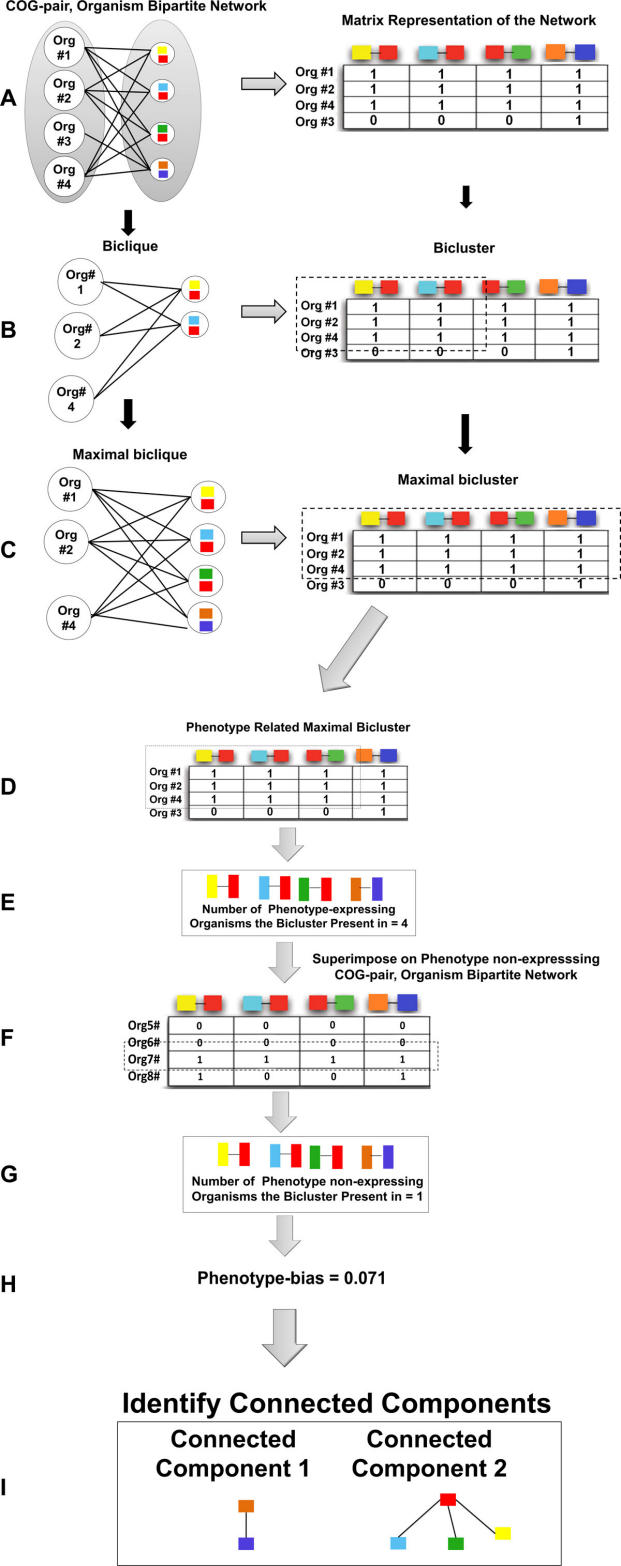
**Methodology overview to identify phenotype-biased functional modules**.

In this paper, we propose a methodology (Figure 1 and Figure 2) to identify the statistically significant functional modules that are phenotype-biased. Phenotype-biased means that it is more conserved across phenotype-expressing organisms and less conserved across phenotype non-expressing organisms. The functional modules are identified by a comparative analysis using both phenotype-expressing and phenotype non-expressing organisms. The structure of the functional modules is a subgraph that is a connected component which is then filtered to identify the statistically significant components. The method does not require parameters similar to the parameters *α *and *β*, in [2] to decide the number of organisms the resulting subsystem should be present in.

In our earlier conference paper [14], we identified the biclusters common to a set of phenotype expressing organisms and analyzed these biclusters for potential cross-talking pathways. In this paper, we extend the methodology to utilize phenotype non-expressing organismal information to identify phenotype-biased functional modules. This increases the confidence in the phenotype-relatedness of the identified modules. The phenotype-bias is quantified using the hypergeometric statistical test. In [14], we performed the analysis only for the hydrogen production phenotype. Here, we have also analyzed respiration, gram-positivity, gram-negativity and motility phenotypes.

## Results

### Experimental setup

We set up experiments with four different phenotypes, hydrogen production (dark and light fermentation), respiration, gram-stain, and motility. The organisms for each phenotype were identified using literature search [15,16]. The functional association network for each organism was obtained from the STRING [1] database and the edge score cutoff used was 700 (termed as *high confidence *[1]).

### Hydrogen production

Biological hydrogen production is being looked at as a source of alternate energy and there are plenty of microorganisms that can utilize different organic substrates to produce hydrogen. This makes it a useful alternate energy option to explore [17-19]. Identifying cellular subsystems related to hydrogen production will be extremely useful to genetic engineers looking to make the process of biological hydrogen production more efficient. The light and dark fermentation are two important sub-phenotypes of hydrogen production and experiments based on these phenotypes have been discussed in this section.

#### Light fermentation

Initial review of the light fermentation clusters shows the presence of a set of 13 identical COGs found across all 8 COG clusters. These "core" COGs include genes necessary for synthesis of hydrogenase complex(es).

Nitrogen-fixation is the process, in which nitrogenase catalyzes the conversion of nitrogen gas to ammonia and inadvertently results in the production of hydrogen gas as a byproduct [20,21]. Two COGs (COG2710 and COG1348), which are associated with the expression of two key proteins, nitrogenase iron protein (NifH) and molybdenum iron protein [20], were present across all the clusters. Although, the presence of these two proteins is essential for nitrogen-fixation to be carried out by light fermenting microorganisms, expression of various genes in other metabolic pathways plays important roles in either directly or indirectly regulating the expression of genes encoding NifH proteins. These proteins include ferric iron regulation proteins (sigK, clpB, and fur-related), ammonia ligase (glnA), and nitrogenase [22]. In this study, glutamate ammonia ligase (glnA), a key gene for nitrogenase (NifH), and genes encoding proteins for iron uptake, are assembled in the same cluster. In *Anabaena*, iron uptake proteins and some nitrogen proteins (e.g., Ntc) have been shown to regulate genes encoding glutamate synthetase (glnA) [23]. Review of the role of glutamine synthetase in *Anabaena *indicates that this enzyme is responsible for regulating nitrogenase activity, thus impacting hydrogen production [23]. The indirect regulation of nitrogenase by iron uptake proteins provides an example of cross-talk between iron and nitrogen-related metabolic pathways.

In addition to nitrogenase, proteins associated with the synthesis of uptake or expression of hydrogenase, were identified in 11 of the 19 COGs present in Table 1. Hydrogen uptake proteins help with removing excess hydrogen to maintain the reducing environment in cells [24]. We also identified a number of proteins (e.g., Hyd and Hyp) involved in formation of [NiFe]-uptake hydrogenases. The presence of maturation hydrogenase factors (COG0068, COG0298, COG0309) and accessory proteins for uptake of nickel (COG0378) are consistent with literature reports describing the structure of hydrogenase complexes. Inclusion of hy-drogenase proteins in Table 1 is likely due to the relationship of hydrogenase proteins with iron uptake genes. To function properly, iron is needed to form the NiFe center present in the large hydrogenase subunit (HupL) [25]. As such, hydrogenase maturation is dependent on cross-talks with iron uptake.

**Table 1:** COGs associated with light fermentation identified by the method

COG ID	COG Description
COG0068	Hydrogenase maturation factor
COG0298	Hydrogenase maturation factor
COG0309	Hydrogenase maturation factor
COG0374	Ni,Fe-hydrogenase I large subunit
COG0375	Zn finger protein HypA/HybF(possibly regulating hydrogenase expression)
COG0378	Ni2+-binding GTPase involved in regulationof expression and maturation of urease and hydrogenase1
COG0409	Hydrogenase maturation factor
COG0680	Ni,Fe-hydrogenase maturation factor
COG1740	Ni,Fe-hydrogenase I small subunit
COG0174	Glutamine synthetase
COG0535	Predicted Fe-S oxidoreductases
COG0716	Flavodoxins
COG1348	Nitrogenase subunit NifH (ATPase)
COG2082	Precorrin isomerase
COG2710	Nitrogenase molybdenum-iron protein,alpha and beta chains
COG2370	Hydrogenase/urease accessory protein
COG1941	Coenzyme F420-reducing hydrogenase, gamma subunit
COG3259	Coenzyme F420-reducing hydrogenase, alpha subunit
COG0735	Fe2+Zn2+ uptake regulation proteins

In previous studies by Lopez-Gollomon [23], the nitrogen regulator protein NtcA was found to work together with the iron-uptake protein, Fur, to co-regulate genes involved in various metabolic functions. Metabolic functions co-regulated include the transcriptional regulation protein and glutamine synthesis [26]. In this study, genes encoding iron uptake regulator proteins (COG0735) were clustered together with genes encoding glutamine synthetase (COG0174). The co-appearance of these two COGs suggests the possible cross-talk between iron uptake and ammonia assimilation networks. In addition, there is indication that hydrogenase proteins, such as HupUV, are involved in regulating the glutamine synthetase gene, glnAII, in some organisms [20,27].

#### Dark fermentation

Unlike light fermentation, we did not observe a large set of COGs present across all clusters. For this set of organisms, only two COGs were identified as present across all clusters. This may be partially due to the following two reasons. First, the selection of species and species diversity have some impact on the types of clusters generated. Second, dark fermentation organisms tend to utilize a greater variety of fermentation pathways, such as acetate fermentation and butyrate fermentation pathways [28]. Greater variation in fermentation routes will not produce as large of a "core" set of COGs across all clusters.

An example of COG clusters identified in dark fermentative bacteria is present in Table 2. In this cluster, 13 different COGs consisting of proteins that are either directly or indirectly responsible for the uptake or production of hydrogen, are present. Of these COGs, 7 are related to the synthesis or expression of [NiFe]-hydrogenase, an enzyme that catalyses the reversible oxidation of molecular hydrogen, and plays a vital role in anaerobic metabolism [27]; the others are involved in nitrogen and iron metabolic pathways that include proteins like nitrogenase, iron uptake proteins, such as Fur (COG0735), ammonia assimilation proteins, such as glutamine synthetase (COG3968), and proteins involved in electron transfer. Previous findings by Butland *et al*. [29] show that the presence of proteins (e.g., HypE, HypD, HupS, HupD) is typically associated with hydrogen uptake [25,30]. Based on the other genes (e.g., hybG, hupS) present in the cluster, we can predict that [NiFe]-hydrogenase is associated with hydrogen uptake in this group of organisms.

**Table 2:** COGs associated with dark fermentation identified by the method

COG ID	COG Description
COG0298	Hydrogenase maturation factor
COG0309	Hydrogenase maturation factor
COG0374	Ni,Fe-hydrogenase I large subunit
COG0409	Hydrogenase maturation factor
COG0680	Ni,Fe-hydrogenase maturation factor
COG1740	Ni,Fe-hydrogenase I small subunit
COG0535	Predicted Fe-S oxidoreductases
COG1348	Nitrogenase subunit NifH (ATPase)
COG2710	Nitrogenase molybdenum-iron protein, alpha and beta chains
COG0716	Flavodoxins
COG0735	Fe2+/Zn2+ uptake regulation proteins
COG2082	Precorrin isomerase
COG3968	Uncharacterized protein related to glutamine synthetase

In addition to hydrogenase maturation and expression proteins, Fe-S oxidoreductases were identified. As part of the structure of [NiFe]-hydrogenase, Fe-S metal centers are located on the small subunit of the hydrogenase complex [25,27]. Thus, it is expected that iron uptake pathway would cross-talk with hydrogenase-related pathways. Furthermore, the iron uptake pathway also cross-talks with nitrogen metabolism, in a sense that iron uptake proteins can be involved indirectly in nitrogen metabolism through regulation of nitrogenase and maintaining the reducing environment in the cell through hydrogen uptake (hydrogenase) [26,31].

It has been shown that cross-talk between iron uptake and nitrogen metabolism enables regulation of ammonia assimilation [21]; it may be possible that the uncharacterized glutamine synthetase protein in Table 2 is subject to such regulation. In our results, the gene encoding the uncharacterized glutamine synthetase proteins was only present in a few species, including *Clostridium acetobutylicum *and *Clostridium beijerinckii*, which both contained nitrogenase and hydrogenase enzymes. It has been demonstrated that, in light fermenting organisms, such as *Rhodopseudomonas palustris*, glutamine synthetase is regulated by hydrogenase accessory proteins (HupUV) [21]. However, to the best of our knowledge, this relationship has not been described in dark fermentation organisms. This knowledge increases the probability that the uncharacterized glutamine synthetase protein maybe present in the COG cluster oweing to its association with nitrogenase proteins, which may further indicate a possible cross-talk between ammonia assimilation and nitrogen metabolism.

### Motility

The motility experiment was set up with a set of 85 motile and 56 non-motile organisms chosen from Slonim *et al *[16]. The method identified clusters that contained COG1360, COG1558, COG1157, COG1684, and COG1536 (Table 3). All these COGS are related to flagella proteins. The flagella proteins are those that enable the organisms to move. The method also found COG0643, COG0835, and COG0784 that are related to bacterial chemotaxis. It is well known that chemotaxis controls an organism's movement with respect to the chemical composition of its environment. For example, it helps the organism moves to the areas where there is very high concentration of food [32].

**Table 3:** COGs associated with motility identified by the method

COG ID	COG Description
COG1843	Flagellar hook capping protein
COG1291	Flagellar motor component
COG1344	Flagellin and related hook-associated proteins
COG1256	Flagellar hook-associated protein
COG1338	Flagellar biosynthesis pathway, component FliP
COG4786	Flagellar basal body rod protein
COG1360	Flagellar motor protein
COG1558	Flagellar basal body rod protein
COG1157	Flagellar biosynthesis,type III secretory pathway ATPase
COG1684	Flagellar biosynthesis pathway, component FliR
COG1536	Flagellar motor switch protein
COG1766	Flagellar biosynthesis/type III secretory pathway lipoprotein
COG1684	Flagellar biosynthesis pathway,component FliR
COG1987	Flagellar biosynthesis pathway,component FliQ
COG1338	Flagellar biosynthesis pathway, component FliP
COG1886	Flagellar motor switch/type III secretory pathway protein
COG0643	Chemotaxis protein histidine kinaseand related kinases
COG0835	Chemotaxis signal transduction protein
COG0784	FOG: CheY-like receiver
COG0643	Chemotaxis protein histidine kinase and related kinases
COG1508	DNA-directed RNA polymerase specialized sigma subunit, sigma54 homolog
COG1191	DNA-directed RNA polymerase specialized sigma subunit

COGS related to the Type III secretion system (COG1766, COG1684, COG1987, COG1338, and COG1886) were also identified. It has been shown that Type III secretion proteins share similarities with flagella proteins in structure and function [33]. Additionally, we identified COG0835, COG0643, COG1344, COG1291,COG0784, COG1508, and COG1191 associated with the two-component systems. This is a signaling pathway that regulates motility [34].

### Respiration

This experiment was set up with a set of 77 aerobic organisms and 57 anaerobic organisms. For aerobic respiration, COGs related to the enzymes present in the TCA cycle were identified. They are COGs related to citrate synthase (COG0372), acitonase (COG1048), and Malate dehydrogenases (COG0039) (Table 4). Some COGs such as the malate synthase (COG2225), isocitrate synthase (COG2224), glyoxylate bypass were also found. The entire list of TCA-related COGs identified can be found in Table 4. There were also other literature verified COGs (COG0843, COG0109,COG1048, COG1622, COG1845, and COG0372) found by the method described in [35].

**Table 4:** COGs associated with aerobic respiration identified by the method

COG ID	COG Description
COG0372	Citrate synthase
COG1048	Aconitase A
COG0045	Succinyl-CoA synthetase, beta subunit
COG0074	Succinyl-CoA synthetase, alpha subunit
COG0479	Succinate dehydrogenase/fumarate reductase,Fe-S protein subunit
COG1053	Succinate dehydrogenase/fumarate reductase,flavoprotein subunit
COG2142	Succinate dehydrogenase,hydrophobic anchor subunit
COG0039	Malate/lactate dehydrogenases
COG2224	Isocitrate lyase
COG2225	Malate synthase
COG2084	3-hydroxyisobutyrate dehydrogenaseand related beta-hydroxyacid dehydrogenases
COG2379	Putative glycerate kinase

For anaerobic experiment, we found COG1924, COG1592, COG2221, and COG2033. The COG1924 is related to oxygen sensitive proteins [36] (Table 5). The other COGs were pulled out computationally by another genotype-phenotype methods [35,36] applied to the anaerobic phenotype. We also identified COGs from the Arginine and proline metabolism, the reason for this could be attributed to the L-argnine which could serve as an energy source for anaerobes.

**Table 5:** COGs associated with anaerobic respiration identified by the method

COG ID	COG Description
COG1924	Activator of 2-hydroxyglutaryl-CoA dehydratase(HSP70-class ATPase domain)
COG1592	Rubrerythrin
COG2221	Dissimilatory sulfite reductase(desulfoviridin), alpha and beta subunits
COG2033	Desulfoferrodoxin

### Gram-positive and gram-negative

This experiment was set up with a set of 61 gram positive bacteria and 109 gram negative bacteria. For gram negativity, COG2877, COG2885, COG1044, COG1519, COG0763, and others related to the Lipopolysaccha-ride biosynthesis were found (Table 6). This pathway has been shown to be related to gram-negativity [33]. Another set consisting of COG0043, COG0163, COG2227, COG1008, and COG1005 were found. These are associated with the ubiquinone pathway that is also shown to be associated with gram-negativity [33]. The COG0848 found by the method has been shown to be associated with the target phenotype [37].

**Table 6:** COGs associated with gram negativity identified by the method

COG ID	COG Description
COG2877	3-deoxy-D-manno-octulosonic acid(KDO) 8-phosphate synthase
COG2885	Outer membrane protein andrelated peptidoglycan-associated (lipo)proteins
COG1044	UDP-3-O-[3-hydroxymyristoyl]glucosamine N-acyltransferase
COG1519	3-deoxy-D-manno-octulosonic-acid transferase
COG0763	Lipid A disaccharide synthetase
COG0838	NADH:ubiquinone oxidoreductase subunit 3 (chain A)
COG0337	3-dehydroquinate synthetase
COG0852	NADH:ubiquinone oxidoreductase 27 kD subunit
COG1143	Formate hydrogenlyase subunit 6/NADH:ubiquinone oxidoreductase 23 kD subunit (chain I)
COG0713	NADH:ubiquinone oxidoreductase subunit 11 or 4L (chain K)
COG0649	NADH:ubiquinone oxidoreductase 49 kD subunit 7
COG0382	4-hydroxybenzoate polyprenyltransferaseand related prenyltransferases
COG0043	3-polyprenyl-4-hydroxybenzoate decarboxylaseand related decarboxylases
COG0163	3-polyprenyl-4-hydroxybenzoate decarboxylase
COG2227	2-polyprenyl-3-methyl-5-hydroxy-6-metoxy-1,4-benzoquinol methylase
COG1008	NADH:ubiquinone oxidoreductase subunit 4 (chain M)
COG1005	NADH:ubiquinone oxidoreductase subunit 1 (chain H)
COG1663	Tetraacyldisaccharide-1-P 4'-kinase
COG0774	UDP-3-O-acyl-N-acetylglucosamine deacetylase
COG1212	CMP-2-keto-3-deoxyoctulosonic acid synthetase
COG0859	ADP-heptose:LPS heptosyltransferase
COG2908	Uncharacterized protein conserved in bacteria
COG2870	ADP-heptose synthase, bifunctionalsugar kinase/adenylyltransferase
COG3307	Lipid A core - O-antigen ligase and related enzymes
COG0445	NAD/FAD-utilizing enzymeapparently involved in cell division
COG0848	Biopolymer transport protein

From gram-positive bacteria (Table 7), the method identified COG3764 and COG3773. COG3674 relates to plasma membrane proteins and was identified by previous research as related to gram-positivity [37]. The COG3773 is associated with endospore formation that usually occurs in gram-positive bacteria when there is a lack of nutrients. We also found COG0619 and COG1122 as single connected component; these COGs are associated with uptake of MET/or MET-precursors, which are associated with regulation of genes involved in amino acid metabolism in gram-positive bacteria [38].

**Table 7:** COGs associated with gram positivity identified by the method

COG ID	COG Description
COG3764	Sortase
COG3773	Cell wall hydrolyses involved in spore germination
COG0619	ABC-type cobalt transport system, permease component CbiQ and related transporters
COG1122	ABC-type cobalt transport system, ATPase component

## Discussion

In summary, the proposed methodology identifies the phenotype-biased cellular subsystems. The results of the method provide clues on which subsystems are co-present to help in phenotype expression. This information could potentially be put to use by genetic engineers. However, there are three points that should be pondered over.

There are several points that are worth bringing out especially when analyzing network data of both phenotype-expressing and phenotype non-expressing organisms. The first point is on the importance of phylogenetic diversity of the underlying organisms. Our results depend on the organisms chosen for the experiment. There could be cases where the conserved biclusters were identified purely due to the fact that the chosen organisms were phylogentically close. Thus, as part of future work we will look into incorporating some scoring mechanism, such that the identified phenotype-biased biclusters are also conserved across a set of phylogenetically diverse set of organisms.

The second point of discussion stems from the fact the same organism can express multiple phenotypes. Currently we look at only one phenotype at a time. The current methods that analyze multiple phenotypes at the same time [33] do so by looking at one phenotype at a time and by correlating the results of the different experiments. Analyzing multiple (possibly related) phenotypes together might provide new insights into pathway cross-talking mechanisms. Another direction of future work is to extend our method to work with multiple phenotypes.

The third point of discussion stems from the fact that a phenotype may have several subphenotypes. For example, all hydrogen producing organisms do not express the phenotype in the same manner. Hydrogen production has three subphenotypes dark fermentation, light fermentation and biophotolysis, two of those were discussed in this paper. Thus, when methods seek to identify phenotype-related systems using multiple organism data, it is imperative to identify systems present in any subset of organisms as opposed to all the organisms. The subsystem present in a subset (as opposed to all) may be a specific path (subphenotypes) to carry out the phenotype. Our methodology models that intuition by using the bicluster definition for the functional module, thus naturally allowing the identified cluster to be present in any organism subset (of size ≥ 2).

## Conclusion

We have developed a method to identify phenotype-biased functional modules by utilizing both phenotype-expressing and phenotype non-expressing organismal network data. By applying our method to four phenotypes, hydrogen production, gram stain, motility and respiration, we were able to identify functional modules that were associated with the target phenotypes. The findings were validated via literature evidence.

## Methods

### Orthologous group-pair, organism bipartite network

In order to identify functional modules across a given set of organismal protein functional association networks, we need a representation that would help us enumerate these modules efficiently. The organismal protein functional association network is obtained from STRING database [39], each node is a protein and a pair of proteins are connected by an edge if there is some evidence of their functional association. Some examples of the evidences considered in STRING are gene fusion, co-occurrence on the same operon, co-expression etc. In this paper we propose the *orthologous group-pair*, *organism bipartite network *that combines the information present in all of the individual organismal protein functional association networks into one single network (Figure 1).

As a first step to constructing this network, we need some kind of transformation that would help us understand the commonalty and differences among the networks. One such transformation is replacing all proteins in all of the organismal networks with their corresponding orthologous group IDs (Figure 1.2). The most common representation used in biology is the manually curated *Clusters of Orthologous Groups (COGs) *[40].

In the second step, we construct two sets, *O *and *C *(Figure 1.3). In *C*, each element is a pair (*x*, *y*), where both *x *and *y *are *COGs*. In *O*, each element represents an organism. These two sets become the two partites of the graph.

As a final step, we construct the orthologous group-pair, organism bipartite network (Figure 1.3), *N *= (*O*, *C*, *E*). An edge (*a*, *b*) ∈ *E*, where *a *∈ *O *and *b *= (*u*, *v*) ∈ *C *exists if and only if the COG pair (*u*, *v*) is functionally associated in organism *a*, i.e., in the organismal protein functional association network *A *= (*V*(*A*), *E*(*A*)) corresponding to organism *a*, ∃*x*, *y *∈ *V*(*A*) : *x *and *y *belong to orthologous cluster groups *u *and *v*, respectively, and (*x*, *y*) ∈ *E*(*A*). Since in this paper we make use of COGs, the network *N *will henceforth be referred to as the ***COG-pair, organism bipartite network***.

### Network representation and preprocessing

The COG-pair, organism bipartite network, *N *is represented using an adjacency matrix for the purpose of identifying the conserved functional modules (Figure 1.4). The organisms are the row-headers and each column header is a COG-pair. A matrix cell has a 1, if the corresponding organism (row-header) and the COG pair (column-header) are connected by an edge in network *N*. This matrix is typically sparse.

### Obtaining the conserved COG clusters

As a first step to identifying the modules, we identify sets of COG edges that are conserved across two or more organisms. These sets can be represented as bicliques (Figure 2.B) in the COG-pair, organism bipartite network. To avoid enumerating the same information more than once, we only enumerate the maximal bicliques (Figure 2.C).

**Definition 0.1 ***Given a bipartite graph N *= (*O*, *C*, *E*), *a subgraph S *= (*O*', *C*', *E*') *of N is a biclique if *∀*a *∈ *O*' *and b *∈ *C*', (*a*, *b*) ∈ *E*'.

**Definition 0.2 ***A biclique S of N is also maximal if there is no supergraph S*' *of S that forms a biclique in N*.

The problem of identifying maximal bicliques using the binary matrix representation translates to identifying the maximal biclusters (Figure 2.B) in the matrix. Although any biclustering technique that works on binary matrices would suffice, we chose Prelic *et al.'s *Bimax biclustering algorithm [41]. There are two reasons for this choice: (1) Bimax performs on par with the best biclustering techniques [41], and (2) It has also been shown that Bimax is able to output all the optimal (maximal) biclusters in the given binary matrix [41]. The algorithm uses a divide-and-conquer approach to enumerate maximal bicliques (maximal biclusters) in the COG-pair, organism bipartite network (Figure 2.C).

### Comparative analysis using phenotype non-expressing organisms

So far, we have hypothesized that biclusters conserved across 2 or more phenotype-expressing organisms are likely phenotype related. We strengthen the notion of *phenotype-related *to *phenotype-biased *by performing comparative analysis using a set of both phenotype-expressing and phenotype non-expressing organisms. Phenotype-biased biclusters are likely to be conserved more across a set of phenotype expressing organisms and less across a set of phenotype non-expressing organisms.

Figure 2.D-G shows the steps in the comparative analysis pipeline. The analysis begins with a set of biclusters identified in Figure 2.C. The orthologous group-pair, organism bipartite network is built for the set of phenotype non-expressing organisms. This network is then converted into its matrix representation (phenotype non-expression matrix). Each phenotype-related bicluster, identified previously, is now analyzed in the context of the phenotype non-expression matrix. We seek to identify the number of phenotype non-expressing organisms the bicluster is conserved in. This information can be utilized to calculate the phenotype-bias of this bicluster.

The phenotype-bias is quantified by using the hypergeometric statistical test. Let *P *be the total number of organisms (both phenotype-expressing and phenotype non-expressing). Let *S *be the total number of phenotype expressing organisms. Let *X *be total number of organisms (both phenotype-expressing and phenotype non-expressing) the bicluster *B *is present in. Let *Y *be the number of phenotype-expressing organisms the bicluster *B *is present in. The bias (*p*-value) of the bicluster *B *is calculated as follows:

(1) $$bias(B)=\frac{\left({}_{Y}^{S}\right)*\left({}_{X-Y}^{P-S}\right)}{\left({}_{X}^{P}\right)}$$

We apply a *p*-value cutoff of 0.05 to identify all the phenotype-biased biclusters.

### Enumerating the connected components

Each phenotype-biased maximal bicluster identified in the previous section represents the set of COG-COG edges conserved across the set of phenotype-expressing organisms. However, we cannot consider the COG-COG edge set as a functional module as is. A functional module has to be a connected subgraph of an organismal network as opposed to a collection of edges. A connected component subgraph is one where there is path between every pair of nodes in the subgraph. However, there is no guarantee that all the COG-COG edges in the bicluster are connected. Thus, all the connected component subgraphs from the COG-COG edge set of each bicluster are enumerated (Figure 2.I).

### Assessing statistical significance

The results of the previous section only guarantee that the subgraphs output are connected components but there is no clear indication whether the subgraphs could potentially represent functional modules or if their occurrence was purely random. One way to check this would be to compare the density of each component with the density that could be obtained at random for a subgraph with the same number of nodes.

The Monte Carlo method [42,43], a robust statistical significance method, is utilized to assess the significance. For every connected component *S *= (*V*, *E*), we calculate the density *β*(*S*). We randomly sample subsets of |*E*| COGs each from the set of all possible COGs *M*. We estimate an empirical *p*-value as *R/W*, where *W *is the total number of random subsets generated (*W *~ 1000) and *R *is the number of random subsets that produce a test statistics *β*() greater than or equal to that of *β*(*S*). We then use a cutoff (say 0.05) to identify the statistically significant components.

## Competing interests

The authors declare that they have no competing interests.

## Authors' contributions

KP and KW developed and implemented the computational model and the algorithm and conducted computational experiments. AR, KG, and KP provided biological validation. KP, KW, KG, and AR provided the initial draft of the manuscript. JM suggested and supervised the study related to the hydrogen production from wastewater and waste materials. NS provided the problem statement, supervised the development of the computational methodology, and provided suggestions on methodology validation. JM and NF contributed to preparing the final version of the manuscript. All authors have read and approved the final manuscript.
